# Cesium could be used as a proxy for potassium in mycorrhizal *Medicago truncatula*

**DOI:** 10.1080/15592324.2022.2134676

**Published:** 2022-10-19

**Authors:** Arjun Kafle, Kevin Garcia

**Affiliations:** Department of Crop and Soil Sciences, North Carolina State University, Raleigh, North Carolina, USA

**Keywords:** Arbuscular mycorrhizal symbiosis, cesium, *Medicago truncatula*, potassium, *Rhizophagus irregularis*

## Abstract

Arbuscular mycorrhizal (AM) fungi interact with the roots of most land plants and help them to acquire various mineral resources from the soil, including potassium (K^+^). However, tracking K^+^ movement in AM symbiosis remains challenging. Recently, we reported that rubidium can be used as a proxy for K^+^ in mycorrhizal *Medicago truncatula*. In the present work, we investigated the possibility of using cesium (Cs^+^) as another proxy for K^+^ in AM symbiosis. Plants were placed in growing systems that include a separate compartment only accessible to the AM fungus *Rhizophagus irregularis* isolate 09 and in which various amounts of cesium chloride (0 mM, 0.5 mM, 1.5 mM, or 3.75 mM) were supplied. Plants were watered with sufficient K^+^ or K^+^-free nutrient solutions, and shoot and root biomass, fungal colonization, and K^+^ and Cs^+^ concentrations were recorded seven weeks after inoculation. Our results indicate that Cs^+^ accumulated in plant tissues only when K^+^ was present in the nutrient solution and when the highest concentration of Cs^+^ was used in the fungal compartment. Consequently, we conclude that Cs^+^ could be used as a proxy for K^+^ in AM symbiosis, but with serious limitations.

Potassium (K^+^) is a macronutrient required by all living organisms. In plants, K^+^ represents 2 to 10% of the dry biomass, and the optimum cytoplasmic K^+^ concentration for enzymatic activities is around 100–200 mM.^1^ Maintaining an optimum K^+^ concentration in plant cells is thus essential for the efficiency of several physiological processes, including plasma membrane polarization, stomatal aperture regulation, the acquisition of other nutrients, and ultimately, plant growth.^2–4^ Although K^+^ ions are typically abundant in soils, only two fractions are immediately available to plants: K^+^ in water solution and K^+^ in an exchangeable form. Together, these two fractions only represent 2.2% of the total soil K^+^.^5^ Thus, depending on the soil type, the concentration of plant-available K^+^ can range from 0.1 to 1 mM and often leads to the development of K^+^ depletion zones around plant roots.^6,7^ Consequently, plants have developed efficient strategies to improve soil K^+^ uptake, including the expression of high-affinity transport proteins and the establishment of beneficial associations with microbes, such as arbuscular mycorrhizal (AM) fungi.^8^

Although multiple studies reported that plants colonized by AM fungi accumulate more K^+^ than non-colonized ones,^9–14^ tracking K^+^ transport from the fungus to the roots remained challenging since K^+^ isotopes have a short half-life or are expensive. Recently, we demonstrated that rubidium (Rb^+^), a classical proxy for K^+^, can be used to track K^+^ movement in mycorrhizal associations.^15,16^ In the present study, we report that cesium (Cs^+^) could be used as another proxy for evaluating K^+^ transport in the model legume plant *Medicago truncatula* colonized by the AM fungus *Rhizophagus irregularis* isolate 09, but with serious limitations.

Custom-made two-compartment systems containing one root compartment (RC) and one fungal compartment (FC) were used, as described previously.^15,16^ Two 52-micron meshes were placed between the compartments, allowing the fungal hyphae to colonize the FC from the RC, but not the roots. Two-week-old *M. truncatula* (A17 accession) seedlings were transferred into each RC filled with pre-washed Turface® and inoculated or not with 400 fungal spores along with some colonized root segments of root organ cultures. Plant RCs were watered with either K^+^-deprived (-K, 0 mM) or K^+^-supplemented (+K, 3.25 mM) Long Ashton solutions. The FCs were provided with 20 ml of 1/10 strength of the corresponding nutrient solution once a week along with milli-Q water between weekly nutrient solution additions. Ten days before harvest, FCs were provided with 20 ml of CsCl at various concentrations (0 mM, 0.5 mM, 1.5 mM, or 3.75 mM) every 2 days, while the RCs were still watered with the -K or +K solutions until harvest at 7 weeks. Around 0.2 ppb of Cs^+^ was detected in the Turface®, which is far less than what was added in the FCs and detected in plant tissues (see below).

Shoot and root tissues were collected and oven-dried after taking a subsample of fresh roots to check the AM colonization (Figure 1). The shoot and root dry weights of mycorrhizal plants (AM) growing in +K condition were significantly higher than non-mycorrhizal (NM) plants or AM plants in -K condition (Figure 1a,b). Under K^+^-deprived conditions, no significant differences in shoot dry weights were noted between NM and AM plants (Figure 1a). However, root dry weights of AM plants at -K were significantly higher than NM plants growing in the same condition (Figure 1b). We also recorded that an average of around 30–40% of *M. truncatula* roots were colonized by the AM fungus, without any significant differences within each treatment (Figure 1c). We also observed that the amount of Cs^+^ supplied in the FCs did not affect plant biomass and root colonization in any K^+^ availability. Shoot K^+^ and Cs^+^ concentrations were also quantified using ICP-OES or ICP-MS, respectively, at the Environmental and Agricultural Testing Services (EATS) at North Carolina State University (Figure 2). AM plants displayed significantly greater shoot K^+^ concentration than NM plants only when K^+^ was provided in the watering solution (Figure 2a). This observation was not made in the absence of K^+^ supply, indicating that the fungus had a positive impact on plant K^+^ nutrition, as already reported in other studies.^11,12,14,16^ Concerning shoot Cs^+^ concentration, no difference was found between all AM and NM plants in K^+^-deprived condition (Figure 2b). At +K, significantly higher shoot Cs^+^ concentration was detected only in AM plants when 3.75 mM of CsCl was added to the FCs (Figure 2b). Indeed, we did not observe any difference in shoot Cs^+^ concentration between AM and NM plants when the FCs were provided with less than 3.75 mM of CsCl (Figure 2b).

**Figure 1.**
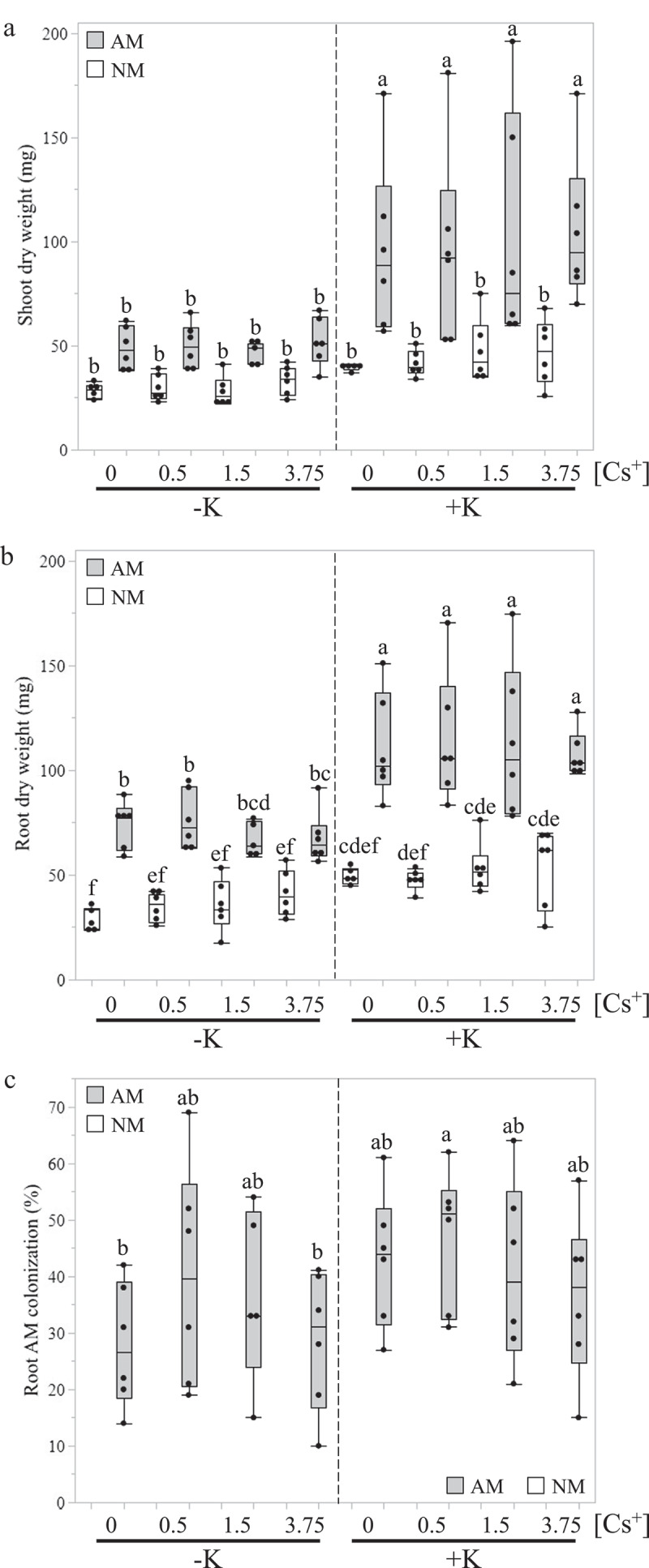
Biomass and root colonization of *Medicago truncatula* plants inoculated by *Rhizophagus irregularis* isolate 09 under potassium-deprived and -sufficient conditions, and with 0 mM, 0.5 mM, 1.5 mM, or 3.75 mM of cesium chloride added to the fungal compartment. Shoot (a) and root (b) dry weights were determined in seven-week-old *M. truncatula* plants inoculated (AM) or not (NM) by the AM fungus *R. irregularis* isolate 09 in deprived (-K, 0 mM) or sufficient (+K, 3.75 mM) K^+^ conditions. (c) The rate of fungal colonization was determined on *M.truncatula* roots grown under -K or +K conditions after seven weeks of co-culture using the grid line intersection method. In FCs, 0 mM, 0.5 mM, 1.5 mM, or 3.75 mM of CsCl was added during each of the four watering sessions ([Cs^+^]) ten days before harvest. Different letters indicate significant differences among all possible combinations of the treatments according to ANOVA followed by LSD post hoc tests (P < 0.05), n = 5–6.

**Figure 2.**
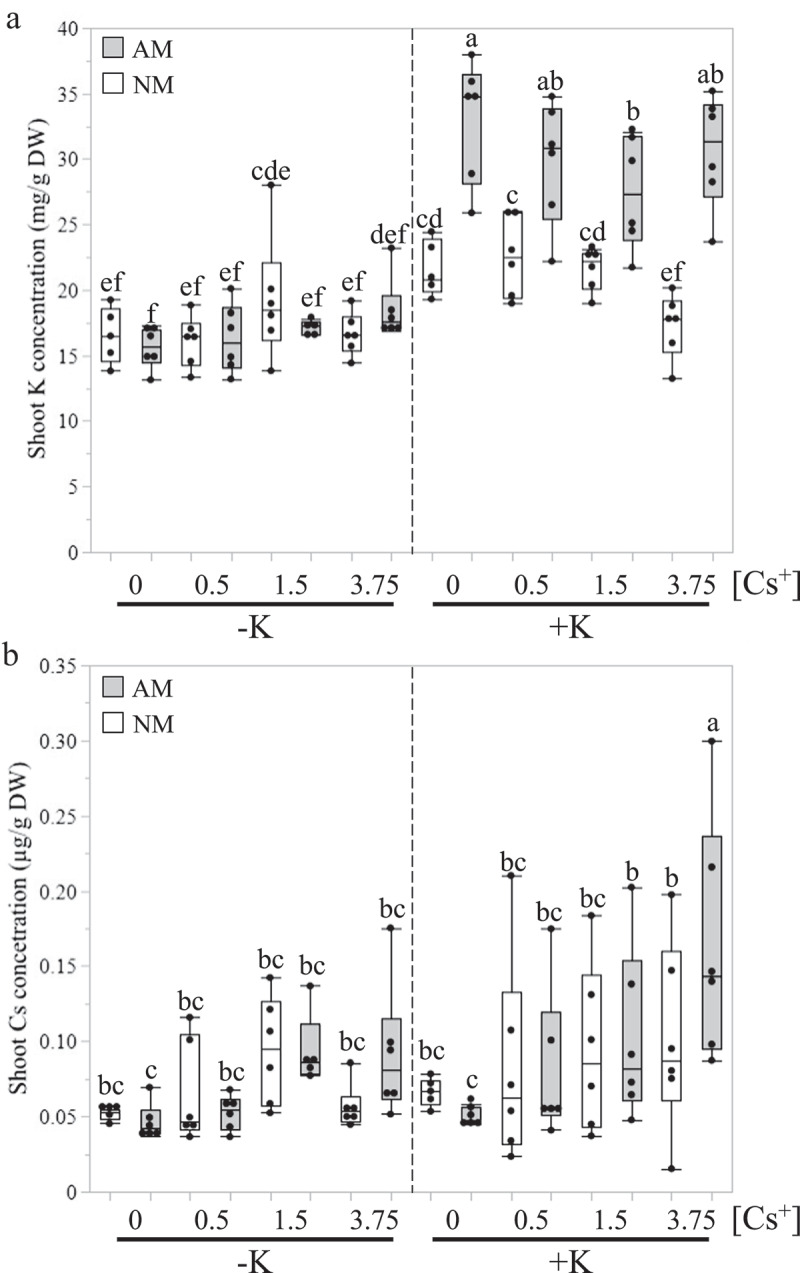
Shoot potassium and cesium concentrations in *Medicago truncatula* inoculated by *Rhizophagus irregularis* isolate 09 under potassium-deprived and -sufficient conditions and with 0 mM, 0.5 mM, 1.5 or 3.75 mM of cesium chloride added to the fungal compartment. Potassium (K, a) and cesium (Cs, D) concentrations were determined by ICP-OES or ICP-MS in the shoots of seven-week-old *M. truncatula* plants inoculated (AM) or not (NM) by the AM fungus *R. irregularis* isolate 09 in deprived (-K, 0 mM) or sufficient (+K, 3.75 mM) K^+^ conditions. In FCs, 0 mM, 0.5 mM, 1.5 mM, or 3.75 mM of CsCl was added during each of the four watering sessions ([Cs^+^]) ten days before harvest. Different letters indicate significant differences among all possible combinations of the treatments according to ANOVA followed by LSD post hoc tests (P < 0.05), n = 5–6.

These data revealed that Cs^+^ could be used as a proxy to track K^+^ movements in AM *M. truncatula*, but with less efficacy than Rb^+^. Indeed, even if more Cs^+^ was detected in the shoot of AM plants compared to NM ones, this difference was spotted only at the highest level of CsCl provided to the fungus and at high K^+^. Using a similar setup, we recently showed that a lower Rb^+^ supply (1.5 mM) can be used to track K^+^ in AM *M. truncatula* plants whatever the external K^+^ concentration.^16^ Additionally, the shoot Cs^+^ concentrations found here (around 0.1 μg/g of dry weight) were 200 times lower than the Rb^+^ concentrations we reported previously (around 20 μg/g of dry weight), making the detection of Cs^+^ more difficult than Rb^+^ in plant tissues. This lower detectable concentration can be due to the larger molecular weight and ionic radius of Cs^+^ ions compared to K^+^ and Rb^+^ ions, which may affect its transport upon AM symbiosis.^17^ Indeed, although some reports have demonstrated that AM fungi can transfer Cs^+^ from the soil to colonized plants,^18–20^ others reported the absence of Cs^+^ transport in mycorrhizal plants.^21^ Additionally, some authors mentioned that exposure to high Cs^+^ concentrations could inhibit the AM colonization in *M. truncatula*, although we did not find such reduction here.^22^ They also reported that Cs^+^ transport to the host plant was not consistent and could depend on the experimental factors such as plant and fungal species, soil types, as well as external K^+^ and Cs^+^ concentrations.^22^ It is also worth noting that Cs^+^ is a classical blocker for K^+^ channels and could even inhibit plant growth at high concentrations.^23–25^ We did not observe any biomass reduction in our experiment, probably due to the short exposure to Cs^+^ (10 days) and because the plants were not in direct contact with Cs^+^ ions. Finally, some reports described that AM fungi could accumulated radioactive Cs^+^ in their tissues, limiting its transport to host plants.^19^ Therefore, it is possible that most Cs^+^ we added in the FCs were stored in fungal hyphae and not transferred to *M. truncatula*, except when the highest level of Cs^+^ (3.75 mM) was supplied.

To conclude, we propose that Cs^+^ could be used as a proxy to track K^+^ movements in comparative experiments between AM and NM plants, but serious limitations come with this approach, including the very low detection of Cs^+^ ions in plant tissues, the impossibility to evaluate the absolute transport of K^+^, and the need to use higher amounts of Cs^+^ than Rb^+^. Consequently, Rb^+^ should be preferred over Cs^+^ as a proxy for K^+^ in mycorrhizal symbioses.
